# Myocardial ASL-CMR perfusion imaging with improved sensitivity using GRAPPA

**DOI:** 10.1186/1532-429X-18-S1-P100

**Published:** 2016-01-27

**Authors:** Terrence R Jao, Hung P Do, Krishna S Nayak

**Affiliations:** 1 grid.42505.360000000121566853Biomedical Engineering, University of Southern California, Los Angeles, CA USA; 2 grid.42505.360000000121566853Department of Astronomy and Physics, University of Southern California, Los Angeles, CA USA; 3 grid.42505.360000000121566853Electrical Engineering, University of Southern California, Los Angeles, CA USA

## Background

Myocardial arterial spin labeling (ASL) is a non-contrast CMR technique for measuring myocardial blood flow (MBF), but has relatively low signal-to-physiological noise (PN). Therefore perfusion measurements are made over several averaged pixels within left ventricular (LV) segments and not individual pixels. Shorter image acquisition times using SENSE parallel imaging has been shown to significantly reduce PN and improve sensitivity to MBF. However, SENSE is unable to reconstruct images at a field of view (FOV) smaller than the object being imaged and requires a large FOV, which leads to fewer number of pixels for averaging in the LV.^1^ In this study, we demonstrate that GRAPPA parallel imaging (rate 1.6), which has no such FOV restrictions, reduces PN even further than SENSE (rate 2), despite having a longer image acquisition window.

## Methods

Six healthy volunteers were scanned using a 3T GE Signa Excite HD scanner with an 8-channel cardiac coil. Myocardial ASL measurements were made at a single mid short axis slice using flow alternating inversion recovery (FAIR) ASL and snap shot balanced SSFP imaging. Images were accelerated using either SENSE with a reduction factor of 2 (96 × 48) or GRAPPA with a reduction factor of 1.6 (96 × 60, 24 ACS lines). This corresponded to 153 ms and 192 ms imaging windows respectively. The minimum FOV that caused no aliasing was chosen for SENSE imaging while the minimum FOV that causes no aliasing within the left ventricular myocardium was chosen for GRAPPA imaging^1^. 6 breath-held labeled/control image pairs were acquired for each image acceleration scheme. MBF, PN, and temporal SNR (TSNR = MBF/PN) were measured within the left ventricular myocardium ROI.

## Results

GRAPPA and SENSE accelerated ASL were acquired at a FOV of 20.1 ± 2.8 cm and 26.6 ± 3.1 cm with 133.6 ± 35.7 pixels and 67.6 ± 13.5 pixels within the segmented LV respectively. Average per-segment MBF and PN measurements across all subjects from GRAPPA and SENSE were 1.97 ± 0.44 ml/g/min and 1.78 ± 0.73 ml/g/min with a corresponding TSNR of 5.44 and 3.26 respectively. No significant difference in MBF was found between the two methods (p = 0.3230) while PN found in GRAPPA was significantly lower than that found in SENSE (p = 0.0011). Global and per segment MBF, PN, and TSNR are summarized in table 1.

**Table 1:** Comparison between GRAPPA and SENSE accelerated cardiac ASL

		Global				Per-segment			
	FOV (cm)	MBF (ml/g/min)	PN (ml/g/min)	TSNR	N	MBF (ml/g/min)	PN (ml/g/min)	TSNR	N
GRAPPA	20.1 ± 2.8	1.73 ± 0.22	0.21 ± 0.10	11.48 ± 8.85	532.0 ± 132.7	1.97 ± 0.85	0.44 ± 0.22	5.44 ± 3.04	133.6 ± 35.7
SENSE	26.6 ± 3.1	1.75 ± 0.85	0.60 ± 0.34	3.41 ± 1.38	269.6 ± 43.2	1.78 ± 1.04	0.73 ± 0.55	3.26 ± 3.15	67.6 ± 13.5
p-value	0.0003	0.9681	0.0289	0.0086	0.0061	0.3230	0.0011	0.0013	2.6 × 10-12

Global analysis was performed on the entire left ventricular myocardium. Per-segment analysis divided the myocardium into 6 regional segments. Values are reported as mean ± standard deviation across all subjects. (FOV) field of view, (MBF) myocardial blood flow, (PN) physiological noise, (TSNR) temporal signal-to-noise, (N) number of pixels within each segment.

## Conclusions

We demonstrate that GRAPPA parallel imaging has lower PN and higher TSNR than SENSE parallel imaging while having similar estimated MBF, despite having a longer imaging window. This is because GRAPPA is able to image at a lower FOV, which increases the number of pixel for averaging and decreases partial voluming of the blood pool at the myocardial borders. Further reductions to PN are expected by reducing the imaging window by combining GRAPPA with partial Fourier reconstruction or with faster imaging acquisition schemes such as EPI.

